# Internet Use and Its Impact on Engagement in Leisure Activities in China

**DOI:** 10.1371/journal.pone.0089598

**Published:** 2014-02-21

**Authors:** Ronggang Zhou, Patrick S. W. Fong, Peking Tan

**Affiliations:** 1 School of Economics and Management, Beihang University, Beijing, P. R. China; 2 Department of Building & “Real Estate”, Hong Kong Polytechnic University, Hong Kong, P. R. China; 3 Research & Development, Greater China, Millward Brown, Beijing, P. R. China; NIDDK/NIH, United States of America

## Abstract

**Introduction:**

Internet use has become an increasingly common leisure time activity among Chinese citizens. The association between Internet use and engagement in leisure activities is especially unclear among China population. This study aims to investigate Internet usage and to determine whether active Internet use is a marker for low or high levels of leisure time activities.

**Methods/Principal Findings:**

With the use of a face-to-face structured questionnaire interview, a total of 2,400 respondents who met all screening requirements were surveyed to answer the questions in eight major cities in China. 66.2% (*n* = 1,589) of all respondents were identified as Internet users. Of these Internet users, 30.0%, 24.1%, 26.4%, and 19.6% were clustered as “informative or instrumental users,” “entertainment users,” “communication users,” and “advanced users,” respectively. Regarding time spent on Internet use in leisure time, more than 96% reported going online in non-work situations, and 26.2% (*n* = 416) were classified as “heavy Internet users.” A logistic regression analysis revealed that there were significant differences in some leisure activities between non-Internet users and Internet users, with an observed one-unit increase in the leisure time dependence category increasing the probability of engaging in mental or social activities. In contrast, Internet users were less engaged in physical exercise-related activities. In addition, advanced Internet users were generally more active in leisure time activities than non-Internet users and other types of users.

**Conclusion/Significance:**

Internet use is one of very common leisure activities in Chinese citizens, and age, gender, income, and education are the key factors affecting Internet access. According to different types of leisure activities, Internet usage has different impacts on leisure activity engagement. High Internet dependence has no significant negative influence on engagement in mental or social leisure activities, but this group respondent tended to be less engaged in physical activities.

## Introduction

Leisure activity can be defined as the voluntary use of free time for activities outside the daily routine, and it is one of the major components of a healthy lifestyle [1]. Engagement in leisure activities provides opportunities to meet life values and needs and contributes to subjective well-being [2], [3], [4], [5]. Currently, understanding and making better use of the Internet to improve users’ quality of life is an important research focus [6]. The Internet continues to be used worldwide and has changed the pattern of life in recent decades.

According to the 31^th^ statistical report on Internet development, China’s Internet users, who aged at six or above and have used Internet within past six months, included 564 million users by the end of 2012. Of these users, the largest group is made up of urban residents (72.4%) [7]. Internet use has become an increasingly common leisure time activity in this population. Unfortunately, Internet use, especially with regard to sociability, has been thought to be inversely linked to quality of life [3]. One of potential reasons for this negative effect on quality of life is the imbalanced allocation of time between Internet use and other regular leisure activities. By changing Internet use patterns and spending suitable amounts of time on regular leisure activities, quality of life may be enhanced [3], [8], [9]. Although a significant body of research has focused on understanding this issue, the association between Internet use and leisure activity engagement is still a controversial issue. Therefore, it is important to understand Internet usage and then to determine whether active and high Internet use is a marker for low or high levels of regular leisure time activities.

To answer this question, we must first understand the digital divide that is characteristic of Internet use. The use of the Internet bridges previously reported digital divide gaps, which can be illustrated by investigating the systematic differences in terms of socioeconomic variables, demographic variables, or socio-environmental factors between Internet users and those who do not access the Internet frequently. Broad survey studies confirm that the previously cited gaps are quickly disappearing [10], [11]. However, the digital divide, in terms of demographic, socioeconomic, and educational differences in the access to and usage of the Internet, is still important for understanding Internet usage, especially in developing countries.

A related area of Internet use that has attracted investigators’ attention is Internet use patterns and users’ typology with respect to their use pattern. Several studies have demonstrated that people with similar levels of access engage the Internet in fundamentally different ways [12], [13]. The issue is related to the user’s Internet usage pattern involves two important variables–online activities and time spent online. The increasing number of Internet users who spend more time online and engage in increasingly diverse activities has captured the attention of policy makers and social researchers [6]. According to previous studies [7], [14], [15], web motives or online activities vary in many ways, including informative activities, social or communicatory activities, transaction activities, and entertainment activities. By integrating the degree of Internet access and online activities, Internet user types can be identified. For example, those who access the Internet with a very varied and broad Internet behavior have been termed “advanced users”, those who have the highest mean scores in goal-oriented activities (e.g., searching for information about goods or services) have been labeled as “instrumental users”, those who have the highest access Internet with regards to enjoyment activities such as downloading games or music have been clustered as “entertainment users,” and those who have occasional or no Internet access have been categorized as “sporadic users” or “non- users” [16]. Internet user types can be a useful way to describe Internet use patterns.

Although little research has systematically focused on the relationship between Internet use and leisure activity engagement, there is evidence that suggests the impact of Internet use on user activities in their leisure time. Based on the psychological perspective, leisure behaviors have a positive impact on cognitive function and dementia based on their physical, mental, or social aspects. Therefore, we tend to accept the classifications of “physical activity,” “mental activity,” and “social activity” [1]. With respect to the impact of the Internet on users’ social lives, major contradictory findings have been reported. For example, some research shows that increased Internet usage has been associated with a decline in users’ interactions with family members within the household and a reduced social circle [8], [17]. In contrast, other studies have suggested that the Internet may have less of an impact on many aspects of social life than is frequently supposed and can actually enhance the social lives of its users [14], [18], [19], [20]. With regard to the association between Internet use and physical activity, leisure time physical activity levels were largely independent of Internet and computer use among a sample of adults with an average age of 45 years [21].

Despite efforts to understand the relationship between Internet use and leisure time social activities and physical activities, the cumulative results from these studies suggest that the situation is complex and controversial. In summary, (a) The time spent between Internet use and leisure activities is a key factor for investigating the impact of Internet use on leisure activities, especially when considering the engagement or dependence on the Internet for leisure purposes. However, few studies have been systematically conducted to examine the association between leisure time Internet use and other leisure behavior. (b) The disagreements above may result from group or individual differences. Recently, Internet users’ typologies have attracted investigators’ attention [16]. The Internet “means different things to different people and is used in different ways for different purposes” [22], and we believe that Internet use by different types of users with different purposes will have mixed impacts on other leisure activities.

To our knowledge, few studies have evaluated how leisure time Internet use and Internet user characteristics (e.g., user topology, gender, and age group) relate to other leisure time activities. The current study seeks for the first time to examine the associations between Internet use, specifically in leisure time, and leisure time activities in a large socially diverse sample of Chinese citizens. Thus, the aims of the present study were to 1) understand the Internet usage pattern in Chinese citizens, especially to address the factors that affect Internet access among this population; 2) investigate whether leisure time Internet use or dependence affects engagement in other leisure activities, and specifically in different gender and age groups; 3) compare the engagement in leisure activities among Internet user types including non-Internet users. In relation to healthy lifestyles and quality of life, this information will help provide direct evidence to clarify the controversial results in the field.

## Research Methodology

### Ethics Statement

This study was reviewed and approved by the committee for the protection of subjects at Millward Brown. Written consent was also obtained from each participant before administering the survey according to the established guidelines of the committee. The survey was entered in the records of the National Bureau of Statistics of China.

### Participants

As shown in Table 1, a total of 2,400 respondents completed full interviews and answered the corresponding questions. The respondents were from eight representative cities in China (Beijing, Shanghai, Guangzhou, Chengdu, Shenyang, Wuhan, Xian, and Fuzhou). According to our research design for sampling, the numbers of respondents were balanced well for gender (i.e., 1,200 males and females) and city (i.e., 800 participants who met the requirements were invited to answer the questions in each city). In addition, the participants were approximately balanced among age groups: 14–24 (27.3%), 25–34 (22.6%), 35–44 (22.6%), and 45–60 (27.5%). With respect to educational level, 21.7% of the respondents had a bachelor’s degree or above, 57.5% had a mid-level education (i.e., associate, secondary school, and high school), and 20.8% had less education (i.e., middle school or below). With regard to employee status and income, 16.8% of respondents were students, 16.4% reported no income, 7.2% earned less than 1,000 CNY monthly, 46.8% had a monthly income of between 1,000–2,999 CNY, and 29.6% had a monthly salary of 3,000 CNY or above. Each respondent was approached in a public place such as a supermarket by a trained interviewer. The respondents were informed about the study by the interviewer’s reading of a written introduction, and they were asked to sign an informed consent form if they agreed to complete the survey. During the survey, the interviewers were asked to read each question to the respondents and record the respondent’s answer in a standard format questionnaire. Respondents were ensured that their participation was voluntary and their response would be anonymous. The questionnaire took approximately 20 minutes to complete.

**Table 1:** Comparison of non-Internet users and Internet users with regard to the characteristics of the respondents.

Variables	Total (N = 2400)	N of users (n = 1589, 66.2)^a^	N of non-users (n = 700, 29.2)^a^	Variables	Total (N = 2400)	N of users (n = 1589, 66.2)^a^	N of non-users (n = 700, 29.2)^a^
**Gender** (*χ^2^* = 14.6***)				**Income** (*χ^2^* = 138.1***)			
Male	1,200 (50.0)	839 (69.9)	309 (25.8)	No income	393 (16.4)	304 (77.4)	58 (14.8)
Female	1,200 (50.0)	750 (62.5)	391 (32.6)	Less than 1000 CNY	172 (7.2)	85 (49.4)	82 (47.7)
**Age** (*χ^2^* = 641.4***)				1000–2999 CNY	1,123 (46.8)	655 (58.3)	421 (37.5)
14**–**24 years	654 (27.3)	575 (87.9)	35 (5.4)	3000–4999 CNY	438 (18.3)	326 (74.4)	98 (22.4)
25**–**34 years	543 (22.6)	458 (84.3)	61 (11.2)	5000–6999 CNY	155 (6.5)	120 (77.4)	28 (18.1)
35**–**44 years	542 (22.6)	338 (62.4)	185 (34.1)	7000 CNY or above	115 (4.8)	95 (82.6)	13 (11.3)
45**–**60 years	661 (27.5)	218 (33.0)	419 (63.4)	**City** (*χ^2^* = 39.8***)			
**Education** (*χ^2^* = 438.9 ***)				Beijing	300 (12.5)	208 (69.3)	77 (25.7)
Primary school or below	77 (3.2)	18 (23.4)	59 (76.6)	Shanghai	300 (12.5)	228 (76.0)	60 (20.0)
Middle school	420 (17.5)	172 (41.0)	231 (55.0)	Guangzhou	300 (12.5)	205 (68.3)	82 (27.3)
High school	578 (24.1)	311 (53.8)	233 (40.3)	Chengdu	300 (12.5)	192 (64.0)	98 (32.7)
Secondary school	235 (9.8)	161 (68.5)	57 (24.3)	Shenyang	300 (12.5)	184 (61.3)	94 (31.3)
Associate	566 (23.6)	467 (82.5)	78 (13.8)	Wuhan	300 (12.5)	217 (72.3)	73 (24.3)
Bachelor	486 (20.6)	428 (88.1)	37 (7.6)	Xi’an	300 (12.5)	186 (62.0)	96 (32.0)
Master or above	35 (1.5)	30 (85.7)	4 (11.4)	Fuzhou	300 (12.5)	169 (56.3)	120 (40.0)

***p<0.001.

a Calculations of percentages were based on the total number of respondents involved in responding subcategories.

### Data Collection

Data were gathered with the use of a face-to-face structured questionnaire interview during the month of October 2010 by professional reviewers at Millward Brown. It is a professorial market survey firm, and has affiliates in main cities in China. Thus, the survey were conducted in the eight cities at the same time, and completed in one week. With use of professional methodologies, the requested participants were approached with considering the balance among the main variables of city, gender, and age. The survey contained four sections. The questions were used for the selection of respondents in the first section and included the following criteria: (1) age between 14 and 60 years; (2) no one in the family holding a position in fields such as marketing research, media (TV station/broadcast/newspaper/magazine/Internet), advertising, or public relations; (3) no participation in any survey designed by a marketing research firm in the last three months; (4) have lived in the local city for at least one year. If the respondent candidates were not suitable for any requirements above, the following sections would be ended.

A total of 2,400 respondents who met the requirements were invited to answer the subsequent sections. The second section assessed the respondent’s prior Internet use experiences, including whether they had used the Internet within the last week and within last one month. Then, for those who acknowledged personal Internet use within the last week, questions were asked about the intensity of Internet use average time online for non-work purposes, both during the work week and on the weekends and activities they engaged in while online. The respondents were also asked to select their three favorite online activities. In the next section, the respondents again used the response of “Yes” or “No” to indicate whether they had ever engaged in common activities (e.g., shopping or visiting relatives or friends) during their leisure time within the last month. The final section was developed to establish other demographic characteristics (i.e., educational level, monthly income, etc.).

### Statistical Analyses

All statistical analyses were performed using SPSS version 19.0. First, the respondents were divided into Internet users and non-Internet users and then descriptive statistics and Chi-square tests were applied to analyze the respondents’ demographic and socio-economic characteristics and Internet use. Based on a cluster analysis with respect to leisurely Internet use and online activities, the types of Internet dependence and use were identified. A logistic regression analysis was then used to estimate the odds of reporting Internet use. Finally, we again used logistic regression to adjust for common leisure activities to investigate the impact of Internet use on leisure activity engagement.

## Results

### Demographic Characteristics of Internet Users

As shown in Table 1, regarding Internet use, 66.2% (*n* = 1,589) of all respondents who reported that they had used the Internet within the past week were categorized as “Internet users”, and 29.2% (*n* = 700) of them who responded that they had not used the Internet within the past month were labeled as “non-Internet users.” Because only 4% (*n* = 111) of the respondents reported that they had accessed the Internet one week before during last month, this category of participants was not included in the subsequent analyses. Without adjustment for other variables, Internet use or the lack thereof varied significantly according to a series of demographic measures: gender, age, monthly salary, education level, occupation, and city (*χ^2^*≥14.6, *p*<0.001). The number of family members did not have a significant influence on users’ Internet usage (*χ^2^* = 5.8, *p*>0.05). Additional details for main demographic characteristics are shown in Table 1.

### Patterns of Internet use

Overall, the most common use of the Internet may be for entertainment, including “watching online videos” (59.0% of respondents reported their engagement in this activity within the preceding week, and 30.0% considered it as one of their favorite online activities), “enjoying or downloading songs or movies” (52.9% engagement, and 24.0% reported it as a favorite activity), “playing online games” (40.7% engagement, and 24.6% reported it as a favorite activity), communications with the “use of chat tools or instant messaging” (59.1% engagement, and 39.5% reported the activity as a favorite), and “Email” (40.5% of engagement, and 16.4% of favorites), followed by information surfing activities such as “getting or reading news” (50.5% engagement, and 27.4% of favorites) and “using a search engine to find information” (33.4% engagement, and 12.1% of favorites) and transaction activities such as “buying or selling online” (27.6% engagement, and 9.1% of favorites).

According to [16], Internet user typology reflects not only how different user groups use the Internet in various ways but also how dissimilar is the potential of user types to exploit the benefits of the Internet. To further understand the patterns of Internet use regarding online activities, we conducted a K-mean cluster score analysis to indentify user groups. K-means clustering is a one of methods of cluster analysis, which is the task of grouping a set of objects in such a way that objects in the same group (called cluster) are more similar (in some sense or another) to each other than to those in other groups (clusters).

In this cluster process, responses regarding engagement within the preceding week and favorite were considered together. In the raw data, participants responded “Yes” (coded as 1) or “No” (coded as 0) to engagement and favorite, respectively. First, the raw responses of engagement and favorite were summarized for each online activity. For example, one respondent reported her or his engagement in online videos within the preceding week, and also considered it as one of their favorite online activities, then the new response score is 2; if he or she only reported engagement or considered it as one of them, the new response scored as 1; or scored as 0 for two “No” response. Thus, all variables were within the same range of 0, 1, or 2, and we conducted a K-means score analysis of the transferred data. Then, to determine the number of clusters, we followed the procedures and results suggested by Brandtzæg and Karahasanovic [16]. As a result, except for non-Internet users, we identified four clusters denoting four Internet user types. Table 2 shows the mean score within each cluster. In terms of the user behavior typical of each cluster, we identified the following four types of Internet users. (1) Cluster 1: Advanced users (19.6% of the Internet users). In general, the mean scores of this user type are the highest for almost all Internet variables, indicating an extremely varied and broad Internet behavior. (2) Cluster 2: Informative or instrumental users (30.0% of the Internet users). The mean scores of this cluster are higher than those of the other clusters with regard to getting news. (3) Entertainment users (24.1% of the Internet users). These users have the highest mean scores in goal-oriented activities such as playing online games, watching online videos, and listening to online music. (4) Communication users (26.4% of the Internet users). These users were characterized by the most frequent use of chat tools or instant messaging.

**Table 2:** The percentages of engagement in online activities and favorite activities and the mean score with each cluster designed to assess Internet user typology (n = 1,589).

Activities online	N of engagement reported (%)		N of favorites reported (%)		Cluster^a^							
					1		2		3		4	
***Information activities***												
Get or read news		803 (50.5)	444 (27.9)	**1.17**		**1.08**		0.39		0.53		
Use a search engine to find information		531 (33.4)	193 (12.1)	**1.14**		0.30		0.29		0.27		
Read online digital magazines and books		294 (18.5)	73 (4.6)	**0.6**		0.13		0.17		0.12		
Listen to radio online		78 (4.9)	18 (1.1)	*0.08*		*0.09*		*0.03*		*0.04*		
***Communication activities***												
Send or read e-mail		643 (40.5)	260 (16.4)	**1.28**		0.50		0.27		0.40		
Participate in an online group forum or BBS		205 (12.9)	57 (3.6)	0.52		*0.09*		*0.07*		*0.07*		
Use SNS sites such as Kaixin or Renren.com		239 (15.0)	77 (4.8)	0.32		0.1		0.12		0.14		
Use chat tools or instant messaging		939 (59.1)	627 (39.5)	0.43		0.11		0.18		0.14		
Read or write online blog		345 (21.7)	104 (6.4)	**0.6**		0.21		0.19		0.21		
Use micro-blogging		200 (12.6)	50 (3.1)	**1.32**		0.07		**0.86**		**1.89**		
Make a phone call online		63 (3.9)	9 (0.6)	0.11		*0.03*		*0.04*		*0.02*		
***Transaction activities***												
Buy or sell online		444 (27.6)	145 (9.1)	**0.72**		0.27		0.3		0.29		
Make a reservation online such as for hotel or business		95 (6.0)	11 (0.7)	0.19		*0.06*		*0.02*		*0.03*		
Engage in financial transactions, such as buying or selling stocks		150 (9.4)	94 (5.9)	0.21		0.25		*0.09*		*0.06*		
***Entertainment activities***												
Play online games not including searching for information about games		646 (40.7)	391 (24.6)	**0.67**		*0.09*		**1.88**		0.16		
Watch online videos		938 (59.0)	476 (30.0)	**1.01**		**0.72**		**0.97**		**0.91**		
Enjoy or download songs or movies		841 (52.9)	382 (24.0)	**1.01**		0.59		**0.86**		**0.71**		
***Other activities***												
Seek a job online		113 (7.1)	23 (1.4)	0.23		*0.05*		*0.05*		*0.06*		
Participate in online education (learning on websites that provide educational services)		77 (4.8)	10 (0.6)	*0.16*		*0.04*		*0.02*		*0.03*		
Upload or download files except for music, movies, or TV dramas		418 (26.3)	112 (7.0)	**0.89**		0.13		0.28		0.19		
Engage in e-government activities for complaining, approving, or supervising		34 (2.1)	9 (0.6)	*0.08*		*0.02*		*0.01*		*0.01*		

a Different font styles are used to enhance the readability of the table. Italics are used for cluster means ≤0.10 and bold font is used for cluster means ≥0.60.

The time spent on Internet use during leisure time can reflect the degree of Internet dependence. In this survey, Internet use was frequent in users’ leisure time; more than 96% of respondents reported going online in non-work situations. The percentage of respondents who reported less than four hours per a day was 65.5% and 45.9% on weekdays and weekends, respectively. A total of 26.5% of participants reported that they spent less than ten hours (including four or more hours) per day during weekday leisure time, whereas the percentage was higher on weekends (43.7%). A total of 4.0% and 6.9% of respondents reported ten or more hours of online time on weekdays and weekends, respectively. To describe Internet dependence, we again performed a K-means cluster analysis of the reported average time online per day to establish groups that varied in terms of online time spent in leisure time per day. Before performing this cluster analysis, we first transferred the categories of reported online time per day into absolute online time: 0 hours for “ = 0 h/D,” 2 hours for “(0, 4) h/D,” 7 hours for “(4, 10) h/D,” and 10 hours for “≥10 h/D.” After the transferred values of workdays and weekends were both entered as variables, we used the iterate and classify method and running means for new cluster centers for each iteration. With two clusters designed as the target, Internet users were classified as “regular Internet users” (73.8%, *n* = 1173; the mode category of reported online time were (0, 4) h/D for both workday and weekend) or “heavy Internet users” (26.2%, *n* = 416; the mode category of reported online time were (4, 10) h/D for both workday and weekend).

To help understand whether there is significant influence of demographic variables on Internet usage, we conducted a logistic regression analysis to investigate factors that may predict different types of users. Because the variable number of members in a household was not a significant factor for comparing Internet users and non-Internet users, it was not considered as one of the predictors in the regression models. Table 3 presents the results of the logistic regression analysis, explaining the predictor for the different user types. The particular type of user (i.e., non-user; the type reflecting the aims of Internet use as an advanced user, information user, enjoyment user, or communication user; or the classification reflecting the Internet dependence as a regular user or heavy user) was used as the dependent variable. The independent variables are listed as age, gender, income, education level, and city. During the logistic regressions analyses, non-Internet users’ reference group was Internet user (i.e., coded as 0); and as for one specific user type, all other Internet users were identified as the reference group (for example, information user, communication user, and enjoyment user were all coded as 0 when performing the logistic regressions for advanced users (coded as 1); in the same way, regular user was coded as 0 for estimating the effect of all independent factors on heavy user). For each user type, we report the results from the total sample and the Nagelkerke *R* ^2^ values, which provide an indication of the amount of variation in the dependent variable (user type) explained by the model (from a minimum value of 0 to a maximum of approximately 1). The “odds ratio” values below the independent variables column gives the factor by which the odds of a user belonging to a specific user type increase when the value of a predictor is increased by one code value. This statistic reflects the effect size and the direction of the relationship. As for non-Internet users, all of the independent variables contributed significantly. The model as a whole explained 49% of the variance in non-users. As shown in the table, belonging to an older age category increases the probability by a factor of 1.81. Being female increases the odds of being a non-user by a factor of 1.34. Increasing the income and education level by one code value decreases the probability by a factor of 0.76 or 0.58, respectively. The factor of city was also found as one of significant resources for estimating non-Internet user, the results may indicate that respondents live in lower developed cities tend to report no Internet use (odd rate = 1.10, and generally the developed level of Beijing, Shanghai, and Guangzhou is higher than other cities). As for the four types of Internet activities, all independent variables only account for 4.0% to 15% of the variance among the different user types. Being older increases the probability of being an information user (odd ratio = 1.32), whereas this factor decreases the probability of being one of the other three types of user (odd ratio ≤0.92). Females tend to be communication users more often than males do (odds ratio = 1.75), whereas males tend to be enjoyment users more than females do (odd ratio = 0.53). Increasing the income and education level increases the probability that users will be advanced users (odds ratios were 1.17 and 1.60, respectively), whereas these factors decrease the probability of being enjoyment users (odds ratios were 0.83 and 0.71, respectively). Respondents live in cities with lower developed level tend to be enjoyment user (odd ratio = 1.06), whereas this factor decrease the probability of advanced user (odd ratio = 0.86). As for the types used for categorizing Internet dependence, all independent variables only account for 5% of the variance among regular or heavy users. Being older decreases the probability of being a heavy user (odds ratios = 0.88), and those with a higher education level tend to be heavy users (odds ratios = 1.12).

**Table 3:** Logistic regression analysis (Nagelkerke R2 and Exp (B) with 95% Confidence Interval) with different user types as the dependent variables.

User Types	N (%)^a^	*R* ^2^	Age^b^	Gender^b^	Income^b^	Education^b^	City^b^
Non-Internet Users	700 (29.2)	0.49	1.81(1.70–1.93)***	1.34(1.06–1.67)*	0.79(0.70–0.89)***	0.58(0.53–0.63)***	1.10(1.05–1.63)***
Advanced Users	311 (19.6)	0.15	0.85(0.79–0.92)***	0.79(0.60–1.03)	1.17(1.05–1.31)***	1.60(1.43–1.80)***	0.86(0.81–0.92)***
Information Users	476 (30.0)	0.11	1.32(1.24–1.40)***	1.18(0.94–1.49)	1.06(0.96–1.17)	1.07(0.98–1.16)	1.03(0.98–1.08)
Enjoyment Users	383 (24.1)	0.12	0.91(0.86–0.98)*	0.53(0.42–0.69)***	0.83(0.75–0.93)***	0.71(0.65–0.77)***	1.06(1.01–1.12)***
Communication Users	419 (26.4)	0.04	0.90(0.85–0.96)**	1.75(1.39–2.21)***	0.97(0.88–1.06)	0.95(0.88–1.03)	1.04(0.99–1.09)
Regular Internet Users^c^	1,173 (73.8)	0.05	1.21(1.13–1.29)***	1.14(0.90–1.43)	0.92(0.84–1.01)	0.89(0.82–0.97)**	1.03(0.98–1.09)
Heavy Internet Users^d^	416 (26.2)	0.05	0.83(0.77–0.88)***	0.88(0.70–1.11)	1.09(0.99–1.20)	1.12(1.03–1.22)**	0.97(0.92–1.02)

*p<0.05; **p<0.01; ***p<0.001.

a Calculations of percentages for non-Internet users based on the total number of respondents (N = 2,400); calculations of percentages for other user types based on the number of Internet users (n = 1,589).

b According to the subcategories in Table 1, numbers were used to order corresponding variables of gender (1 = male, 2 = females), age (1 = 14–24 years, 2 = 25–34 years, 3 = 35–44 years, 4 = 45–60 years), income (i.e., monthly salary, 1 = no income, 2 = less than 1000 CNY, 3 = 1000–2999 CNY, 4 = 3000–4999 CNY, 5 = 5000–6999 CNY, 6 = 7000 CNY or above), education (1 = primary school or below, 2 = middle school, 3 = high school, 4 = secondary school, 5 = associate, 6 = bachelor, 7 = master or above) and city (1 = Beijing, 2 = Shanghai, 3 = Guangzhou, 4 = Chengdu, 5 = Shenyang, 6 = Wuhan, 7 = Xi’an, 8 = Fuzhou).

c The mode category of reported online time were (0, 4) h/D for both workday and weekend.

d The mode category of reported online time were (4, 10) h/D for both workday and weekend.

### Leisure Time Internet Dependence and Leisure Activity Engagement

To help understand whether there is a significant difference in leisure activity engagement between non-Internet users and Internet users, a logistic regression model was developed to investigate the predictive effect of leisure time Internet dependence (for non-Internet users, regular Internet users, and heavy Internet users) in terms of other demographic variables (i.e., age, gender, income, and education; since the differences of developed level were not considered carefully in the research design, the factor of city was not considered as one of the predictors in the regression models). As shown in Table 4, each activity was identified as “physical activity,” “mental activity,” and “social activity”. Among all respondents, the five most popular leisure time activities are related to the mental activities: “watching TV” (80.5%), “reading the newspaper” (52.9%), “listening to music” (38.1%), “reading a magazine” (33.7%), and “going shopping” (27.5%). Regarding the effects of Internet dependence, increasing the leisure time dependence category by one code value increases the probability of engaging in three mental activities (i.e., “reading a magazine,” “going to the cinema,” and “going to an amusement park”; odds ratio ≥1.47) and in two social activities (i.e., “singing karaoke with friends,” and “going to a café or bar”; odds ratio ≥1.27). In contrast, Internet users were less engaged in physical exercise-related activities such as “playing sports/physical exercise for health” and “going to a park” (odds ratio ≤0.75). The variables of age, gender, and education level emerged as significant predictors for most leisure activities in all respondents. For more information regarding the differences among these demographic variables, see Table 4.

**Table 4:** The impact of leisure time Internet dependence on leisure activities.

Activities in Leisure Time	Activity type a	N of engagements (%) (N = 2, 400)^b^	N of Non- Users (%) (n = 700)^b^	N of regular users (%) (n = 1,173)^b^	N of heavy users (%) (n = 416)^b^	Logistic Regression analysis (Nagelkerke *R* ^2^ and Exp (*B*) with 95% Confidence Interval) with independent variables					
						*R* ^2^	Age^c^	Gender^c^	Income^c^	Education^c^	Dependence^d^
Watch TV	MA	1933 (80.5)	622 (88.9)	926 (78.9)	312 (75.0)	0.11	1.23(1.16–1.31) ***	1.40(1.12–1.74) ***	1.16(1.06–1.28) **	0.83(0.76–0.89) **	0.99 (0.81–1.20)
Listen to the radio	MA	547 (22.8)	151 (21.6)	295 (25.1)	90 (21.6)	0.01	1.08(1.03–1.14) **	0.96(0.79–1.17)	0.96(0.88–1.10)	1.15(1.07–1.23) ***	1.03(0.87–1.23)
Read the newspaper	MA	1269 (52.9)	385 (55.0)	635 (54.1)	201 (48.3)	0.05	1.16(1.10–1.21) ***	0.99(0.84–1.18)	1.16(1.08–1.25) ***	1.07(1.00–1.13) *	1.05(0.91–1.22)
Read a magazine	MA	809 (33.7)	109 (15.6)	485 (41.3)	178 (42.8)	0.11	0.90(0.86–0.95) ***	1.61(1.34–1.94) ***	1.11(1.02–1.20) *	1.18(1.11–1.26) ***	1.56(1.33–1.83) ***
Go to the cinema	MA	219 (9.1)	17 (2.4)	125 (10.7)	71 (17.1)	0.12	0.79(0.72–0.86) ***	1.46(1.08–2.00) *	1.30(1.16–1.46) ***	1.19(1.06–1.32) **	1.69(1.31–2.19) ***
Go shopping	PA/MA	661 (27.5)	206 (29.4)	311 (26.5)	104 (25.0)	0.04	1.00(0.95–1.05)	2.22(1.83–2.70) ***	1.06(0.98–1.16)	1.02(0.95–0.09)	0.91(0.77–1.08)
Listen to music	MA	915 (38.1)	161 (23.0)	520 (44.3)	176 (42.3)	0.14	0.74 (0.70–0.78)***	1.43(1.19–1.71) ***	1.02(0.94–1.10)	0.98(0.92–1.04)	1.06(0.91–1.25)
See a play, show, or drama	MA	46 (1.9)	17 (2.4)	24 (2.0)	4 (1.0)	0.02^ns^	0.95(0.81–1.11)	0.91(0.90–1.67)	1.24(0.95–1.63)	0.82(0.66–1.02)	0.74(0.43–1.27)
Read books	MA	482 (20.1)	94 (13.4)	282 (24.0)	75 (18.0)	0.05	0.89(0.84–0.94) ***	0.96(0.78–1.19)	0.90(0.82–0.99) *	1.24(1.15–1.34) ***	0.85(0.70–1.03)
Go to an amusement park	MA/PA	126 (5.3)	17 (2.4)	65 (5.5)	33 (7.9)	0.07	0.81(0.72–0.90) ***	2.29(1.53–3.43) ***	1.20(1.02–1.41) *	0.93(0.81–1.06)	1.47(1.06–2.04) *
Go to a general park	PA	613 (25.5)	247 (35.3)	268 (22.8)	68 (16.3)	0.05	1.07(1.02–1.13) **	1.24(1.02–1.51) *	1.00(0.92–1.10)	0.90(0.84–0.97) **	0.74(0.62–0.88) ***
Sing karaoke with friends	SA	286 (11.9)	43 (6.1)	154 (13.1)	72 (17.3)	0.07	0.79(0.73–0.85) ***	1.02(0.79–1.33)	1.28(1.15–1.42) ***	0.96(0.88–1.06)	1.27(1.02–1.60) *
Dine in restaurants	SA	442 (18.4)	104 (14.9)	236 (20.1)	81 (19.5)	0.03	0.97(0.92–1.03)	1.28(1.03–1.59) **	1.23(1.12–1.35) ***	1.12(1.04–1.21) **	1.04(0.86–1.25)
Go to café or bar	SA	95 (4.0)	8 (1.1)	53 (4.5)	33 (7.9)	0.10	0.80(0.71–0.90) ***	0.81(0.53–1.26)	1.55(1.31–1.84) ***	0.90(0.77–1.04)	1.88(1.31–2.69) ***
Visit relatives or friends/Join a party	SA	527 (22.0)	162 (23.1)	261 (22.3)	80 (19.2)	0.02	1.04(0.98–1.09)	1.29(1.05–1.58) *	1.19(1.09–1.31) ***	0.99(0.92–1.06)	0.96(0.81–1.15)
Go on an excursion/Go camping	PA	177 (7.4)	49 (7.0)	87 (7.4)	30 (7.2)	0.04	1.04(0.95–1.13)	1.45(1.04–2.01) *	1.39(1.21–1.60) ***	1.09(0.97–1.23)	0.97(0.74–1.29)
Play sports/Physical exercise for health	PA	374 (15.6)	118 (16.9)	177 (15.1)	57 (13.7)	0.03	0.98(0.92–1.05)	0.75(0.59–0.94) *	1.18(1.07–1.30) **	1.11(1.02–1.21) *	0.75(0.61–0.93) **
Play chess, cards, or mahjong	MA/SA	287 (12.0)	115 (16.4)	126 (10.7)	40 (9.6)	0.08	1.06(0.99–1.14)	0.38(0.29–0.50) ***	1.12(0.99–1.23)	0.81(0.74–0.89) ***	0.87(0.69–1.10)

*p<0.05; **p<0.01; ***p<0.001.

a PA, physical activity; SA, social activity; MA, mental activity.

b Calculations of percentages were based on total number of respondents involved in responding subcategories. Those who accessed the Internet within the past month but did not use in last week were not considered in the table (*n* = 111).

c The way used for coding these variables was the same with that in table 3.

d Non-Internet users were coded as 1, Regular Internet Users was coded as 2, and Heavy Internet Users was coded as 3. And the reference group for “Regular Internet Users” and “Heavy Internet Users” was “Non-Internet Users”.

### Internet Use and Leisure Activities Engagements

With respect to the differences among Internet user types, a logistic regression model was first generated to compare leisure activity engagement between each type of Internet user (i.e., advanced user, information user, enjoyment user, and communication user) and non-Internet user, adjusting for all demographic variables (i.e., age, gender, income, city, and education). We then used the same method to compare leisure activity engagement among the four Internet user types. The results suggested that advanced Internet users were generally more active in leisure time activities than non-Internet users and other user types. In terms of reading books, dining in restaurants, and visiting relatives or friends/joining a party, advanced users reported more engagement than other user types (AOR ≥1.85, *p*<0.01), and no significant differences were observed for engagement in these activities between each of the other three user types and non-users. In contrast, there was no significant difference between advanced Internet users and the other three user types and non-users in the activities of “watching TV”; “seeing a play, show, or drama”; and “playing sports/physical exercise for health.” For the activities of “going to the cinema,” “going shopping,” “going to an amusement park,” “going to a park,” there were no significant differences found between advanced users and enjoyment users. Unlike other activities, the finding also demonstrated that information users were less active in singing karaoke with friends than the other three Internet user types. Additional results are shown in Table 5.

**Table 5:** Estimated Odds of leisure activities based on Internet user type.

Activities in Leisure Time	Advanced Users c(n = 311)		Information Users^c^ (n = 476)		Enjoyment Users^c^ (n = 383)		Communication Users^c^ (n = 419)		Differences among them (AOR)^d^
	n (%)^a^	AOR (95% CI)^b^	n (%)^a^	AOR (95% CI)^b^	n (%)^a^	AOR (95% CI)^b^	n (%)^a^	AOR (95% CI)^b^	
Watch TV	238 (76.1)	1.10 (0.72–1.69)	387 (81.3)	1.04 (0.71–1.51)	303 (79.1)	1.19 (0.79–1.78)	310 (47.0)	0.85 (0.58–1.24)	E>C (1.43 *)
Listen to the radio	98 (31.5)	1.80 (1.24–2.60) **	130 (27.3)	1.42 (1.04–1.92) *	79 (20.6)	1.08(0.76–1.53)	78 (18.6)	0.93(0.66–1.32)	A>C/E (≧1.58 *); I>C (1.61 **)
Read the newspaper	190 (61.1)	1.85 (1.33–2.59) ***	284 (59.7)	1.47 (1.12–1.93) **	180 (47.0)	1.14 (0.85–1.53)	182 (43.4)	0.95 (0.71–1.27)	A>C/E (≧1.68 **); I>C (1.57 **)
Read a magazine	178 (57.2)	5.16 (3.60–7.40) ***	192 (40.3)	2.93 (2.16–3.97) ***	123 (32.1)	2.13 (1.53–2.98) ***	170 (40.6)	2.68 (1.93–3.70) ***	A>I/E/C (≧1.71**); I>E (1.39*)
Go to the cinema	62 (19.9)	3.97 (2.11–7.47)***	45 (9.5)	2.20 (1.19–4.06)*	45 (11.7)	2.88 (1.54–5.37)***	44 (10.5)	2.16 (1.15–4.04)*	A>I/C (≧1.82**)
Go shopping	104 (33.4)	1.38 (0.96–1.97)	115 (24.2)	0.82 (0.60–1.10)	96 (25.1)	0.96 (0.69–1.33)	100 (23.9)	0.76 (0.55–1.06)	A>I/C (≧1.72**)
Listen to the music	154 (49.5)	1.86 (1.31–2.64)***	158 (33.2)	1.17 (0.87–1.59)	188 (49.1)	1.74 (1.28–2.38)***	196 (46.8)	1.51 (1.11–2.05)**	A>I (1.67*); E>I (1.49*)
See a play, show, or drama	9 (2.9)	1.92 (0.66–5.58)	7 (1.5)	0.80 (0.30–2.14)	4 (1.0)	0.52 (0.16–1.73)	8 (1.9)	1.10 (0.40–2.98)	A>E (4.06*)
Read books	114 (36.7)	2.37 (1.60–3.50)***	81 (17.0)	0.99 (0.69–1.43)	69 (18.0)	0.96 (0.65–1.42)	93 (22.2)	1.21 (0.83–1.75)	A>I/E/C (≧2.11***);
Go to an amusement park	31 (10.0)	3.36 (1.59–7.11)**	22 (4.6)	1.62 (0.79–3.29)	26 (6.8)	2.24 (1.11–4.54)*	19 (4.5)	1.24 (0.58–2.63)	A>I/C (≧2.15**)
Go to a general park	77 (24.8)	1.07 (0.74–1.55)	89 (18.7)	0.59 (0.44–0.81)**	92 (24.0)	0.87 (0.63–1.21)*	78 (18.6)	0.64 (0.46–0.90)*	A>I/C (≧1.68**); E>C (1.46**)
Sing karaoke with friends	69 (22.2)	2.47 (1.51–4.07)***	30 (6.3)	0.70 (0.41–1.18)	66 (17.2)	1.98 (1.25–3.15)**	61 (14.6)	1.57 (0.98–2.52)	I<A/E/C (≦0.44***); A>C (1.63**)
Ding in restaurants	98 (31.8)	2.19 (1.48–3.25)***	79 (16.6)	0.96 (0.67–1.37)	59 (15.4)	1.07 (0.72–1.57)	81 (19.3)	1.25 (0.86–1.82)	A>I/E/C (≧1.85**)
Go to café or bar	21 (6.8)	3.45 (1.35–8.79)**	15 (3.2)	1.84 (0.74–4.62)	27 (7.0)	4.29 (1.80–10.21)***	23 (5.5)	3.28 (1.35–7.99)**	E>I (2.27*)
Visit relatives or friends/Join a party	99 (31.8)	2.06 (1.42–2.99)***	84 (17.6)	0.82 (0.59–1.14)	74 (19.3)	1.09 (0.77–1.54)	84 (20.0)	1.08 (0.77–1.53)	A>I/E/C (≧1.96***);
Go to excursion/Go camping	34 (10.9)	1.46 (0.82–2.59)	31 (6.5)	0.80 (0.48–1.35)	24 (6.3)	1.06 (0.61–1.86)	28 (6.7)	0.98 (0.56–1.70)	A>I (1.89*)
Play sports/Physical exercise for health	58 (18.6)	0.79 (0.52–1.22)	62 (13.0)	0.57 (0.39–0.83)***	55 (14.4)	0.72 (0.49–1.08)	59 (14.1)	0.69 (0.46–1.02)	
Play chess, card, or mahjong	43 (13.8)	1.37 (0.85–2.22)	38 (8.0)	0.59 (0.39–0.91)*	54 (14.1)	1.12 (0.73–1.70)	31 (7.4)	0.68 (0.42–1.09)	A>I/C (2.02**); E>I/C (1.64*)

*p<0.05;**p<0.01;***p<0.001.

a Calculations of percentages were based on the total number of respondents involved in responding subcategories. Those who accessed Internet within the past month but did not use in last week were not considered in the table (*n* = 111).

b AOR indicates all odds ratios adjusted for other demographic variables (i.e., age, gender, city, income, and education) and are calculated at a 95% confidence interval.

c The reference group for “advanced users”, “information users”, “enjoyment users”, and “communication users” was “non-Internet users” (i.e., those who have not accessed the Internet within the past month, *n* = 700).

d As for the comparison among the four type Internet users who reported that they use the Internet, the group preceding “>” or “<” was labeled as the reference group.

## Discussion

### Respondent Characteristics and Internet Usage Pattern

In the current study, the main aim was to understand factors and patterns associated with Internet use and their impact on users’ leisure time activities among an urban population in China. More than 66% of the participants answered that they had accessed the Internet in the last week; these respondents were labeled as “Internet users” in this study. Together with those who have not used the Internet within the past week but who have within the past month, the rate of Internet use reached 70% among urban citizens in China. Overall, the results were consistent with the latest CNNIC report, which indicated that 72.4% of urban Chinese people have accessed the Internet in the last six months [7]. Therefore, from a collective perspective, it is reasonable to characterize those who have used the Internet within the past week and those who have not accessed Internet within the past month as “Internet users” and “non-Internet users,” respectively. Investigating how many people use the Internet is very important for understanding the new digital divide, as Brandtzæg et al. addressed in their study [16]. In Europe, a recent survey conducted to understand this issue found that 60% of the population was identified to be either non-users (42%) or sporadic users (18%) [16]. In the US, the Pew Internet and American Life Project represent one of the largest efforts to gather large-scale data on Internet use. This project uses nationwide telephone surveys, most recently in December 2008 (N = 2253). Internet penetration reached 74% for all American adults in 2008, reflecting a sharp increase from 66% 3 years earlier [11]. Compared with these results surveyed in other countries, the digital divide seems smaller in China’s urban population. However, the largest digital divide in China may emerge between urban and rural populations, where the percentage of Internet usage was 72.4% and 27.6%, respectively [7].

Regarding the pattern of the Internet, of particular concern is the proportion of those who engage in different online activities. The number of potential functions of the Internet is substantial, and the activities are diverse. In our study, instant messaging and online video watching are the most prevalent, with nearly 60% of Chinese citizens engaging in these two activities when they access the Internet; over half of our participants reported that they engaged in downloading songs or movies and getting or reading news. Together with the CCNIC report, email, Internet games, searching for information and blogging are currently popular in China [7]. However, there are significant differences in engagement in some online activities between current respondents and other populations. For example, the use of chat tools or instant messaging, email, blogging, and the use of SNS sites have gained ground in communication activities in Chinese citizens. In China, instant messaging remains the most popular Internet activity, and less than 50% of Internet users reported that they use email to contact others. In contrast, in the US, over 80% of online users send and receive email, making email the most popular online activity [11]. With respect to Internet dependence among Internet users, overall, the time of Internet use reported in this survey is higher during weekend leisure time than during workday leisure time. In this survey, we did not identify the purpose of Internet use during leisure time, and most Internet users reported a reasonable time spent online. One successful approach to understanding Internet use patterns is to identify Internet user types [16]. Considering both the responses for activity engagement and favorite activities, we were able to successfully identify five user types: non-users, advanced users, information users, enjoyment users, and communication users. Unlike the term used in a previous study [16], we used the term “communication users” instead of “sporadic users” because email or instant messaging was very popular among our respondents. We used the term “information users” to label information-oriented activities such as getting or reading news and searching for information. By clustering the users’ time spent on Internet use during their leisure time, we also identified three user types to describe their Internet dependence: Non-Users, Regular Users, and Heavy Users. Together with the results of previous studies [12], [16], this categorization is a very useful method by which to distinguish user types for analysis purposes. The two studies also suggested that there may be some differences in terms of the frequency of activity engagement among different survey samples; therefore, it is reasonable and important to use corresponding words to label user types.

A large body of studies suggests that the Internet means different things to different people and is used in different ways for different purposes. A number of factors have been found to relate to Internet access and use, including socioeconomic variables, demographic variables, and education [23]. With regard to the demographic groups, the present results support previous findings (e.g., younger groups are more likely to use the Internet, and the proportion of those who reported that they had not accessed the Internet within the past week decreases with age). The same effect of age on Internet usage was found in the US. The latest figures from adults in a nationally representative sample of US adults showed that 30% of people ages 18–32 use the Internet in comparison with 24% of three other generations of ages 55–63, 64–72, and 73+ [11]. The web continues to be populated largely by younger generations. The “gender gap” in Internet access has been found in a number of previous investigations [23]. The results showed that a greater proportion of Chinese male citizens (70%) accessed the Internet than females (63%). The digital pattern of Internet use is also shaped by socioeconomic status and education. The results from this study show that the prevalence of Internet use in populations with higher economic and education levels is high. Fully 80% of those in higher income brackets (over 5,000 CNY monthly) in major Chinese cities have Internet access, compared with 62% of adults who have lower incomes. In terms of education, more than 80% of Chinese citizens who have higher levels of education (i.e., associate degree or above) are identified as active Internet users in comparison with less than 50% of those with middle or lower level education. These results were consistent with Pew findings [11], which indicate that Internet usage is also relatively well represented across most income and education brackets, although usage increases in relation to annual income and education. Some 95% of Americans who live in households earning $75,000 or more a year use the Internet at least occasionally, compared with 70% of those living in households earning less than $75,000 [24]. In US, the Internet use level is much higher for individuals with a higher level of education (i.e., some college or above) than that for those with a mid-level education (i.e., high school or below) [25]. With consistent results in other populations in developed countries such as the US, the socioeconomic divide with regard to digital access is not likely to close quickly in China, especially among rural citizens.

When different user types are taken into consideration, these digital gaps are changed. For example, older people tend to be information users more often than other user types. The predicting effect of gender is only significant for enjoyment users and communication users. The overall picture is that more males than females tend to be enjoyment users, whereas more females than males tend to be communication users. Those with a higher income and education level tend to be advanced users, whereas those with a lower income and education level tend to be enjoyment users. Regarding time spent on leisure time Internet use, younger users tend to be more dependent on the Internet than older users, and those with higher income and education level also tend to be heavy Internet users more frequently than those with lower income. There is growing evidence that the digital divide in access in terms of gender is closing or has closed as more women begin to use the Internet [26], [27]; however, the gender gap in Internet usage is still present, especially among different user types.

### Internet Use and Engagement in Leisure Activities

In this study, our other main aim was to understand the effect of user leisure time, Internet dependence, and Internet user types on users’ leisure activities. We found that Internet dependence neither decreased nor increased engagement in some mental activities (e.g., watching TV, listening to the radio, reading the newspaper, and going shopping), socially directed activities (e.g., visiting relatives or friends/joining a party and playing chess, cards, or mahjong), and the physical activity of going on excursions or going camping. In contrast, those with higher Internet dependence tend to be more active in interacting with others by singing karaoke and going to a bar or café than non-Internet users and tend to be more engaged in personal promotion or mental activities such as reading a magazine, going to the cinema, and going to an amusement park. Generally, when investigating the effect of Internet use on a life style, these activities could be considered as important indications of the positive impact of personal or social leisure time activities. Consistent with some previous studies [14], [18], [19], [20], our study tends to support the argument that Internet usage contributes to maintaining or increasing many aspects of a citizen’s mental and social activity engagements as the degree of Internet dependence increases. However, Internet users reported less engagement in physical activities such as playing sports/physical exercise for health than non-Internet users did. Jerome and McAuley’s study suggests that efforts to increase personal efficacy in overcoming barriers to exercise may be more practical and have a greater impact on physical activity levels than trying to decrease leisure Internet use, especially among adults [9].

In addition to efforts to investigate the relationship between leisure activities and time spent involved in leisurely Internet use, an examination of the association between leisure activity and the type of Internet use may prove to be more illustrative. In this study, heavy Internet users tended to be less engaged in going to a park than non-Internet users, but the difference was not significant between advanced users and non-Internet users. In addition, the significant difference in the engagement of going to excursions/going camping was only found between information users and non-Internet users. Compared with non-Internet users, time spent on leisure Internet use was not a significant factor for predicting listening to music; however, more advanced users, enjoyment users, and communication users reported more engagement in this activity. Unlike other types of Internet user, more information users tend to be less engaged in the social activities of singing karaoke with friends and going to a café or bar. The reason for this trend may be that older respondents tend to be information users more often than younger respondents do, and engagement in these two leisure activities decreases as age increases. Overall, the current results indicate that advanced users tend to be more active in both regular leisure activities and Internet activities, and in some sense, these engagements are independent of time spent on Internet use. This study provides evidence supporting the importance of identifying Internet user types to investigate the pattern of Internet usage and its impact on respondent’s leisure activities.

### Limitations

Our results should be interpreted with several limitations in mind. First, like many previous studies, a convenience sample was used in this study to recruit respondents. Although we tried to balance the participants in terms of gender, age group, and city, it is very difficult to balance respondents in terms of income level, education level, and occupation. In addition, these conclusions should be understood not to apply to all of China’s subpopulations because there are digital divides regarding Internet use and leisure activities between different groups in China. Especially in accepting the findings regarding city difference should be cautious, since the developed level between the cities were not considered carefully in the research design. Second, there will be some interaction effects in terms of Internet use and leisure activities among types of Internet use, time spent on leisure Internet use (i.e., Internet leisure dependence), and demographics. Third, although we have attempted to identify social, mental, and physical types of leisure activity, each leisure activity actually has social, mental, and physical functions of varying degrees. Further studies may attempt to identify the corresponding role of each leisure activity to improve the systematic understanding of associations between Internet usage and leisure activity.

### Conclusions

In conclusion, the current study indicates the following: 1) Internet use is one of very common leisure activities in Chinese urban citizens; and age, gender, income level, and education level are the key important factors that affect Internet access. 2) Overall Internet usage has different impacts on leisure activity engagement according to the specific type of leisure activity. High Internet dependence has no significant negative influence on mental or social activity engagement, but heavy Internet users tend to be less in engaged in physical activities than non-Internet users. 3) Our study describes an effective method by which to compare leisure activities among different types of Internet users and confirms the argument that Internet use means different things to different people.
